# Retinal upregulation of inflammatory and proangiogenic markers in a model of neonatal diabetic rats fed on a high-fat-diet

**DOI:** 10.1186/1471-2415-13-14

**Published:** 2013-04-15

**Authors:** Jorge E Mancini, Gustavo Ortiz, Juan Oscar Croxatto, Juan E Gallo

**Affiliations:** 1 Nanomedicine & Vision Group, Facultad de Ciencias Biomédicas, Universidad Austral, Pilar, Buenos Aires, Argentina; 2 Department of Ophthalmic Pathology, Fundación Oftalmológica Argentina “Jorge Malbran”, Buenos Aires, Argentina

## Abstract

**Background:**

The contemporary peak of diabetes seems to be related to obesity, sedentary lifestyle and diet. Diabetic retinopathy is the most leading cause of blindness in adulthood in industrialized countries. Our purpose was to evaluate the effect of a high-fat-diet (HFD) on the retina of diabetic rats.

**Methods:**

Two groups of Wistar rats were injected with streptozotocin (STZ) two days after birth using 45 and 90 mg/kg, respectively. At 8 weeks the group on lower doses started to be fed on a HFD. Animals were sacrificed at 37 weeks of diabetes. A control group was made up of non-diabetic rats. Retinal flat mounts were examined using the trypsin digestion technique. Pericytes counts were compared between diabetic and control rats. Cross retinal sections were analyzed by histological techniques and immunohistochemistry and immunofluorescent technique. Primary antibodies against inflammatory and proangiogenic mediators such as RAGE, GFAP, 5-LO, VEGF and TNF-α were used for immunohistochemistry and Western Blot (WB) analyses.

**Results:**

In the two diabetic groups we observed GFAP-positive cells with a morphology and spatial organization similar to those seen in Müller cells. Both diabetic groups had a significantly lower number of pericytes than non-diabetic animals.Increased retinal immunoreactivity of GFAP, RAGE, TNF-α, VEGF and 5-LO was seen in diabetic animals fed on HFD compared to the other groups of animals. WB analysis revealed a higher expression of 5-LO, VEGF, TNF-α and RAGE in the retina of diabetic rats on HFD than in controls and diabetics fed on a normal diet. The percentage of RAGE-stained ganglion cells and ganglion cells was found to be significantly lower in animals on a HFD than in the other animals.

**Conclusions:**

Diabetic animals fed on a HFD showed an increased upregulation of inflammatory and proangiogenic markers. This animal model may be useful to study mechanisms of diabetic retinopathy and therapeutic targets.

## Background

The incidence of diabetes is higher than ever [1]. The contemporary peak of diabetes is related to a higher frequency of obesity and sedentary life as well as a high-fat-diet (HFD) [2]. These facts apply to adults and children alike [3]. For these reasons, several strategies are being used to tackle the problem of obesity and to improve diabetes management [4].

A rise in the incidence of diabetes will cause, in turn, an increase in diabetes complications, such as nephropathy, cardiopathy and diabetic retinopathy, the latter being considered the main cause of new onset blindness in the United States [5].

Advances in molecular biology and medical technology enabled researchers to better understand the early mechanisms of diabetic retinopathy. An increased expression of glial-fibrillary-acidic-protein (GFAP) in the retina and specific abnormalities in the electroretinogram were identified a few weeks after inducing diabetes in animals [6]. These early changes were followed by retinal vascular permeability, microaneurysm development and intraretinal microvascular abnormalities (IRMA) [7].

Type 2 diabetes accounts for 90% of diabetes prevalence [5]. However, most experimental studies on diabetic retinopathy have been done in animal models of type 1 diabetes. In this group of animals an injection of streptozotocin (STZ) is generally used to chemically destroy pancreatic beta cells. Nevertheless, STZ can also be used to develop type 2 diabetes models in which beta cell loss takes place at a slower rate [6-8]. In our study we used the neonatal diabetic rat model fed on a HFD. Animals in this model are treated by an intraperitoneal injection of STZ at day 2 of life and fed on a HFD from week 8 onwards. For comparison purposes we also included a group of animals treated with STZ but fed on a normal diet [9]. Both animal models have been previously characterized [9,10]. To the best of our knowledge, no previous experimental study has investigated the role of HFD in inducing diabetic retinopathy in rats, animals often used in preclinical research. This type of diet is known to accelerate metabolic disorders and their microvascular complications. Bearing in mind the increasing role of obesity and fat food intake in the pathophysiology of diabetes [3,11], we aimed at determining the effect of a HFD on the retina of diabetic rats. Results reported herein were compared to results from non-diabetic and diabetic rats fed on a normal diet.

## Methods

### Rat models of diabetes

Diabetic rats on a HFD. Pregnant Wistar rats provided by Comisión Nacional de Energía Atómica (CONEA, Buenos Aires) were housed in the animal facilities at 21 +/− 1°C on a 12-h light–dark cycle. They were daily examined until delivery. Two days after birth, newborn rats were intraperitoneally injected with STZ (45 mg/kg) (Sigma-Aldrich, St- Louis, MO), in 0.1 ml of 0.1 M solution of citrate buffer of 154 mM of NaCl at pH 4.5 [9,12,13]. The animals remained with their mothers until 21 days of age. Eight weeks after birth, STZ-treated animals were fed with a home-made HFD (25.62% of fat content), (Tables 1 and 2), which was prepared every week and stored at −7°C. Blood samples of 32 μl obtained by tail snipping were utilized to measure blood glucose levels using the Reflotron System (Boehringer Mannheim, Germany) one week after initiating the diet and every month thereafter. Rats which showed glycemic levels >160 mg/dl after one week on the HFD were included in the study.

**Table 1:** Composition of high-fat-diet (HFD) and standard diet

		**Standard**		**HFD**	
		**Total**	**Subtotal**	**Total**	**Subtotal**
Carbohydrates		54.22%		28.86%	
Protein		23.62%		23.60%	
Aminoacids		2.25%		2.25%	
	Lysine		51.11%		51.11%
	Methionine		26.66%		26.66%
	Threonine		22.22%		22.22%
Fat		2.71%		25.62%	
	Saturates		24.22%		46.46%
	Monounsaturated		30.21%		47.51%
	Polyunsaturated		45.6%		6.34%
Ions	Calcium	1.40%		1.60%	
	Sodium	0.05%		0.05%	
	Magnesium	0.18%		0.18%	

**Table 2:** The constitution of fatty acids

**Fatty acids**	**High fat diet**	**Standard diet**
C4:0	0.01	0.04
C6:0	----	0.05
C10:0	0.02	----
C12:0	0.07	----
C13:0	0.02	----
C14:0	3.15	0.53
C14:1 TRANS	0.19	0.13
C14:0 CIS	0.2	0.12
C15:0	0.84	0.15
C15:1	0.49	0.18
C16:0	24.96	15.62
C16:1 TRANS	0.46	----
C16:1 CIS	3.36	1.12
C17:0	2.17	0.45
C17:1 TRANS	0.11	----
C17:1 CIS	0.83	0.17
C18:0	14.9	6.74
C18:1 TRANS	2.84	0.4
C18:1 CIS	38.63	27.73
C18:2 TRANS	0.8	0.08
C18:2 CIS	5	42.12
C20:0	0.1	0.34
C18:3 CIS	0.54	3.4
C20:1	0.6	0.28
C22:0	0.02	0.18
C22:1 CIS	----	0.2
TOTAL	100	100

Diabetic rats fed on a normal diet. Two days after birth animals were intraperitoneally injected with 90 mg/kg SZT (Sigma-Aldrich, St-Louis, MO) in 0.1 M of citrate buffer in 154 mM of NaCl at pH 4.5. Two days after induction blood was drawn intracardially following the method described by Bonner-Weir [9]. Blood glucose was determined in puppies. For the quantification of glycemia, 32 μl of blood and reagent strips (Reflotron System, Boehringer Mannheim, Germany) were used. Animals were labeled as diabetic if they showed hyperglycemia ≥200 mg/dl. Animals remained with their mothers until 21 days of age.

Control rats. Non-diabetic control animals with no STZ injection were fed on standard chow (2.71% of fat content). See Tables 1 and 2.

Ten diabetic rats were sacrificed after 37 weeks of diabetes (45 weeks old in the HFD-SZT group and 37 weeks old in the SZT group with no diet) while ten non-diabetic rats were killed at 41 weeks of age. Animals were handled according to the ARVO Statement for the Use of Animals in Ophthalmic Research.

### Clinical parameters

Each animal was weighed using a standard scale at 8 weeks of life and before death. Lipidemia levels were measured using the Reflotron System every month and before the animal was killed.

### Fatty acid profile

The chemical compound of a HFD and a conventional diet are shown in Table 2. In brief, the HFD contains 25.6% of fat (46.4% of saturated, 47.5% monounsaturated and 6.3% of polyunsaturated fats) while the conventional diet contains 2.71% of fat (24.2% of saturated, 30.2% of monounsaturated and 45.6% of polyunsaturated fats). The diet’s chemical compound was analyzed using animal and vegetable fats and oil-analysis by gas chromatography of fatty acids methyl esters, according to The International Organization for Standardization (ISO 5508: 1990-E) (Table 2).

### Histological examination

Rats were anesthetized by an intraperitoneal injection of 350 mg/kg of chloral hydrate. The eyes were removed and fixed in 4% paraformaldehyde (Sigma-Aldrich, St Louis, MO). Animals were sacrificed by an overdose of chloral hydrate. The anterior segment of the eye and the vitreous were removed and the eye-cup was left one day for fixation. They were then immersed for cryoprotection in 4 concentrations of glucose (5%, 7.5%, 10% and 20%, overnight) and interlocked with resin. Ten-micron cryosections were obtained (Shandon AS325 Retraction) and stained with haematoxylin and eosin (H&E) as well as periodic acid-Schiff (PAS) for microscopic examination using an Eclipse Nikon E800 Microscope (Tokyo, Japan).

### Immunohistochemical and immunofluorescent analyses

The eye was removed and fixed for 48 hours in 4% paraformaldehyde (Sigma-Aldrich, St Louis, MO). The eye-cups were then dissected and fixed in 4% paraformaldehyde in a phosphate buffer for one hour. They were then immersed for cryoprotection in 4 concentrations of glucose (5%, 7.5%, 10% and 20% overnight) and interlocked with resin. Ten-micron sections were obtained and fixed on polylisine-treated glass slides (Shandon AS325 Retraction).

The GFAP expression was analyzed using the primary monoclonal anti-mouse anti- GFAP antibody (BIOGENEX, 4600 Norris Canyon Road, San Ramon, CA, USA). For immunohistochemistry, the sections were first incubated in biotinylated goat-anti-mouse IgG, then in avidin-biotin peroxidase complex Kit and finally in 3.3’-diaminobenzidine (DAB)/nickel solution. For immunofluorescence, axial sections were revealed using the secondary goat-anti mouse antibody with fluorescein. Immunofluorescent analysis was done using the Eclipse Nikon Microscope (Tokyo, Japan).

The following primary antibodies were used for immunohistochemical analyses:

The anti-mouse polyclonal antibody against vascular endothelial growth factor (VEGF) (1:500) (Santa Cruz Biotechnology Inc. 2145 Delaware Avenue Santa Cruz, CA, USA); the anti-mouse polyclonal antibody against receptor advanced-glycation end products (RAGE) (1:300) (AnaSpec Inc, San Jose, CA, USA); the anti-mouse polyclonal antibody against 5-lipoxygenase (5-LO) (1:500) (Assay biotechnology company, San Francisco, CA, USA) and the anti-mouse polyclonal antibody against tumor necrosis factor-alpha (TNF-α) (1:100) (Assay Biotechnology Company, San Francisco, CA, USA). The process for immunohistochemical and immunofluorescent analyses was similar to that described for GFAP.

### Western blot (WB)

Isolated retinas were rinsed in the lysis buffer (5 mM Tris–HCl pH: 6.8, 2 mM MgCl2, 2 mM EDTA, 65 mM NaCl, 1% Triton X-100) and cocktail protease inhibitor (Sigma-Aldrich, St. Louis MO, USA). Protein concentration was determined according to Bradford method [14]. Total protein (10 μg per well) was electrophoresed on a 12% SDS-polyacrylamide gradient gel and blotted onto nitrocellulose. The blot was incubated with primary antibody, washed and further incubated in a secondary antibody. The bands were visualized using the enhanced chemiluminescence detection system (ECL, Amersham, Arlington Heights, IL, U.S.A.). The primary antibodies against 5-LO (Assay Byotech, USA), VEGF (Santa Cruz Biotechnology, CA), TNF-α (Assay Biotechnology Company), RAGE (AnaSpec Inc), β-actin (Santa Cruz Biotechnology, CA) and glyceraldehyde-3-phosphate dehydrogenase (GFPDH) (Santa Cruz Biotechnology, CA) used at 1:1000, 1:500, 1:500, 1:1000, 1:1000 and 1:500 dilutions, respectively, were utilized for WB.

Retinal protein was isolated in pools from four control rats (41 weeks old), diabetic rats on a HFD and diabetic rats on a normal diet (37 weeks of diabetes). At least three independent experiments were performed for each condition.

### Trypsin digestion technique

After the cornea was incised, the eyeball was fixed by immersion for a minimum of 4 hours in 4% formalin buffered with 50 mM Na-K phosphate (pH 7.2). As explained before, the retina was dissected and placed again in 4% buffered formalin for 1 hour more. The retina was cut into a segment convenient for handling and washed overnight in running water. Thereafter, it was incubated at 37°C in a solution of 3% trypsin (Difco 1:250) and 0.1 M tris buffer (pH 7.8) for 1–3 hours. The incubation was finished when the medium became cloudy and the tissue showed signs of digestion. The internal limiting membrane was peeled off in one sheet. The network of vessels was freed of adherent retinal tissue by gentle shaking, mounted on a clean slide and allowed to dry. The preparation was stained with PAS and eosin [15].

### Image analysis

#### Pericyte count

Image analysis measurements were made using a KS400 system (Kontron Elektronic/Zeiss, Eching, Germany) and a Nikon DXM 1200 digital camera (Tokyo, Japan) mounted onto an Eclipse Nikon E-800 microscope for image acquisition. Briefly, images were digitalized in a rectangular frame of 1280 × 960 pixels using the 40× objective in the photo mode of illumination intensity. To adjust for possible defects in the illumination of the optical pathway, a low-pass image was produced for subtraction and background shading correction. After that, a gray value for image segmentation was interactively chosen. In order to define a threshold gray level, all the pixels whose gray value informative content was lower or higher than the segmentation gray were set to white and the others, to black. Pericytes and endothelial cells were counted by an observer in 10 randomly selected sectors of each retina. The number of pericytes was normalized to the relative capillary density (number of cells per millimeter squared of capillary area – 3 pixels^2^ of capillary area = 1 mm^2^). The mean value of pericytes and endothelial cells was calculated in each animal. Samples were evaluated in a masked fashion. We did the same to count acellular capillaries and intercapillary bridges.

Statistical analysis was carried out using one-way analysis of variance and Newman-Keuls Multiple Comparison Post-Test to compare the number of pericytes between diabetic (37 weeks of disease) and control rats (41 weeks of age). All elements out of the two standard deviations were eliminated.

#### Measurement of retinal thickness. Ganglion cell count

Microscopic evaluation of retinas included scanning tissue sections for evidence of gross disease followed by morphometric analysis, which involved measuring retinal thickness and the number of cells in the ganglion cell layer (GCL).

Retinal transversal sections were divided into three equal parts (inner, middle and outer) from the optic disc to periphery. Cells of the GCL were quantified by counting cells in the middle part of the retina. Ten fields (275 μm each of horizontally diameter) were counted. The mean value of these variables was calculated for each animal. Thickness measurements of the entire retina as well as from the inner plexiform layer (IPL) to the photoreceptors layer were taken in the posterior retina from 100 to 500 μm from the optic disc to the periphery, in two different points of each field in each group. Samples were evaluated in a masked fashion.

Image analyses were performed using a Nikon DXM 1200 digital camera (Tokyo, Japan) mounted onto an Eclipse Nikon E-800 microscope for image acquisition. Images were digitalized in a rectangular frame of 1280 × 960 pixels using the 40× and 10 × objectives for GCL cell count and thickness measurements, respectively. To adjust for possible defects in the illumination of the optical pathway, a low-pass image was produced for subtraction and background shading correction.

Statistical analysis was carried out using one-way analysis of variance and Newman-Keuls Multiple comparison Post-Test to compare the number of cells in the GCL between diabetic (37 weeks of disease) and control rats (41 weeks of age). All elements out of the two standard deviations were eliminated.

#### Percentage of ganglion cells stained with RAGE among diabetic rats

Immunohistochemical images of ganglion cells stained with RAGE were digitalized in a rectangular frame of 1280 × 960 pixels using the 20× objective in the photo mode of illumination intensity. Ten images were used for each slide. Four eyes of four animals from each group were analyzed. Twenty slides were utilized in each animal. The percentage of stained ganglion cells was obtained from each slide. Mean and standard deviation were compared between diabetic animals.

## Results

Diabetic rats with and without a HFD had higher glycemia levels (range 160–390 mg/dl) at 37 weeks of diabetes compared to age-matched control rats (range 80–120 mg/dl). Similarly, increased levels of triglyceridemia were observed in diabetic animals (Table 3).

**Table 3:** Weight, glycemia, trygliceridemia and cholesterolemia in diabetic and control rats

**Animal group**	**WA**	**WD**	**N° of rats**	Parameters*			
				Weight**	Gl***	Tri***	Ch***
DBT-HFD	8	0	12	320	111	N-M	N-M
DBT-HFD	9	1	12	340	224	N-M	N-M
DBT-HFD	20	12	12	434	253	139	116
DBT-HFD	45	37	6	449	212	114	115
DBT (2 days old)	0	0	10	7.26	321	N-M	N-M
DBT	37	37	10	388	353	116	121
Control	41	-	12	524	115	91	119

N-M: not measured; WD: weeks on HFD; WA: weeks of age; Gl: Fast glycemia; Tri: triglyceridemia; Ch: cholesterolemia; * Mean ; **gr; *** mg/dl.

Diabetic animals with fasting glycemia below 160 mg/dl were excluded. Control animals with fasting glycemia above 130 mg/dl were not included in the study.

**Figure 1:**
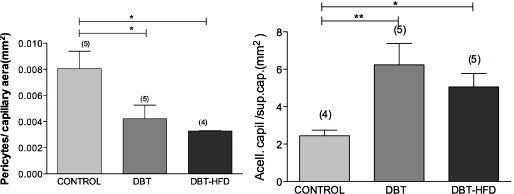
**Pericyte count in diabetic and control rats.** The number of pericytes per capillary area was found to be significantly lower in the retinas of diabetic rats compared to controls but there were no differences between the two diabetic rat groups. The number of acellular capillaries per capillary area was found to be higher in the retinas of both diabetic rat groups compared to controls, but there were no differences between the two diabetic rat groups. The total number of pericytes and acellular capillaries was manually counted in 10 randomly selected fields of each retina. The number of pericytes and acellular capillaries was normalized to the relative capillary density (number per millimeter squared of capillary area – 3 pixels^2^ of capillary area = 1 mm^2^). Samples were evaluated in a masked fashion (***p <0.0001, *p <0.05).

**Figure 2:**
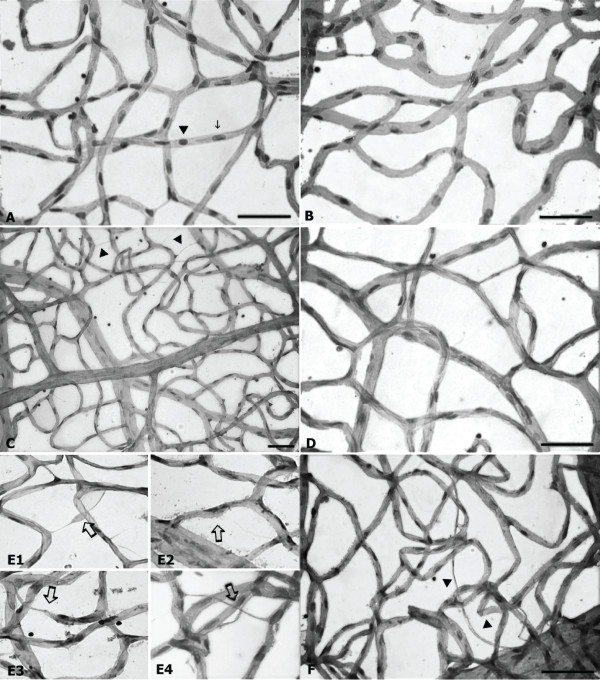
**Histology, using both the trypsin digestion and the conventional technique.** Normal appearance in controls (**A** and **C**), showing intercapillary bridges (**C** arrow head). In diabetics (**B**, **D**, **E** and **F**), acellular capillaries (**F** arrow head and arrows in E1 and E4), capillary obliteration (arrow in E3), vascular collapse and dilation (arrow in E3) in retinas of diabetic rats on a HFD at 37 weeks of disease.

The number of pericytes was found significantly lower in both groups of diabetic rats than in control animals (Figure 1). Vessel dilations, acellular capillaries and capillary obliterations were seen in both diabetic groups (Figure 2).

**Figure 3:**
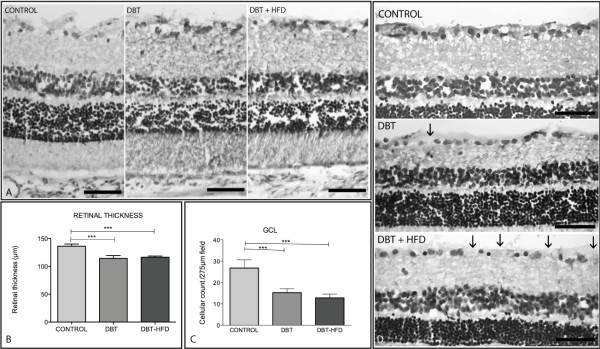
**Retinal thickness (A and B) and number of cells in the ganglion cell layer (GCL) of diabetic and control rat retinas (C and D).** (**A**) The thickness of the entire retina of 4 eyes from each group was measured 10 sections in the posterior retina 100 to 500 μm from the optic disc. Image analysis measurements were taken using a Nikon DXM 1200 digital camera (Tokyo, Japan) mounted onto an Eclipse Nikon E-800 microscope for image acquisition. Barr 50 μm. (**B**) Statistical analysis was carried out using one-way analysis of variance and Newman-Keuls Multiple Comparison Post-Test to compare diabetic groups and controls. All elements out of the two standard deviations were eliminated. (**p <0.001, ***p <0.0001). (**C**) Number of cells in the GCL of diabetic and control rat retinas. The GCL was analyzed in H&E-stained cryosections of diabetic (37 weeks after onset of diabetes) and age-matched control retinas. Samples were evaluated in a masked fashion. Control animals had retinal GCLs with densely packed cells. There was a typically uniform distribution of cells, except for an occasional blood vessel that made a little space between cells (arrows) Barr 50 μm total frame 275 μm. (**D**) Statistical analysis was carried out using one-way analysis of variance and Newman-Keuls Multiple Comparison Post-Test to compare the number of ganglion cells between the groups. All elements out of the two standard deviations were eliminated. (***p <0.0001).

Retinal thickness was reduced in the diabetic groups compared to age-matched controls (Figure 3A,B). Control animals had retinal GCLs with densely packed cells. There was a typically uniform distribution of cells, except for an occasional blood vessel that made a little space between cells (Figure 3C,D), whereas in diabetic rats there was a dropping pattern of ganglion cells from central to peripheral retina. Besides, ganglion cells of both diabetic groups were significantly fewer than in controls (p < 0.001) (Figure 3C).

Unspecified vitreoretinal interphase findings were quite similar in all animal groups (not shown).

**Figure 4:**
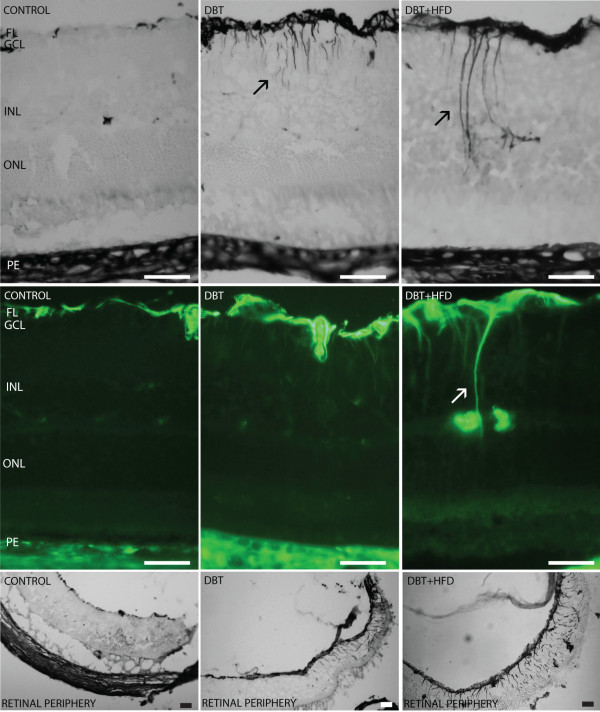
**Immunohistochemical and immunofluorescent expression of GFAP.** Immunohistochemical and immunofluorescent analyses of cross sections of diabetic retinas showed an increased expression of GFAP in cells with a morphology and spatial organization similar to those seen in Müller cells (arrow). GFAP immunoreactivity is higher in diabetic rats than in controls. The expression of GFAP in the peripheral retina was much more extended in diabetic rats fed on a HFD than in rats on a normal diet. Barr 50 μm.

GFAP was expressed in the retinal fiber layer of diabetic and non-diabetic animals. Staining was increased in diabetic rats, but the group fed on a HFD showed more widespread GFAP immunoreactivity in the retinal periphery than did the group fed on a normal diet (Figure 4). In HFD animals GFAP was expressed in cells with morphology and spatial organization similar to those seen in Müller cells (Figure 4).

**Figure 5:**
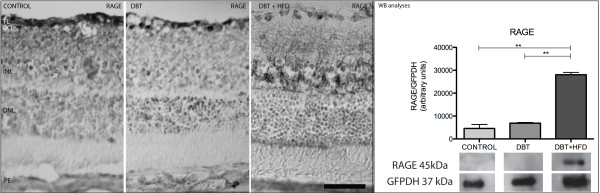
**Immunohistochemical analysis of RAGE expression.** A higher expression was found in diabetic rats than in controls. RAGE expression in the inner nuclear layer (INL) was only observed in diabetic rats on a HFD. Barr 50 μm. Western blot shows a higher expression in diabetic rats on a HFD (**p <0.05).

**Figure 6:**
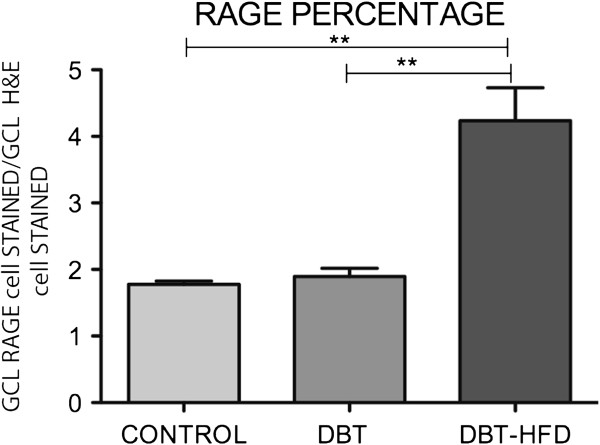
**Percentage of ganglion cells stained with RAGE among diabetic and and non-diabetic rats.** In the middle part of the retina, ganglion cells were counted in histological H&E-stained sections from four animals of each group of diabetic rats and controls. Twenty sections were analyzed in each animal and 10 standard fields (250 μm horizontally) were microscopically examined in each section. Ganglion cells stained with RAGE were then counted, and the percentage of retinal ganglion cells (y-axis) that each sample included was determined. There was a statistically significantly higher rate of ganglion cells stained with RAGE in diabetic rats fed on a HFD compared to diabetic animals fed on a normal diet as well as control rats. (**p < 0.001).

RAGE expression was found to be upregulated in diabetic animals. Its expression at the inner nuclear layer (INL) was only seen in diabetic rats on a HFD (Figure 5). Percentage of ganglion cells stained with RAGE was significantly increased in diabetic rats on HFD compared to diabetic rats fed on a normal diet (Figure 6). Besides, RAGE expression on WB was found to be higher in diabetic animals fed on HFD than in the other groups of rats (Figure 5).

**Figure 7:**
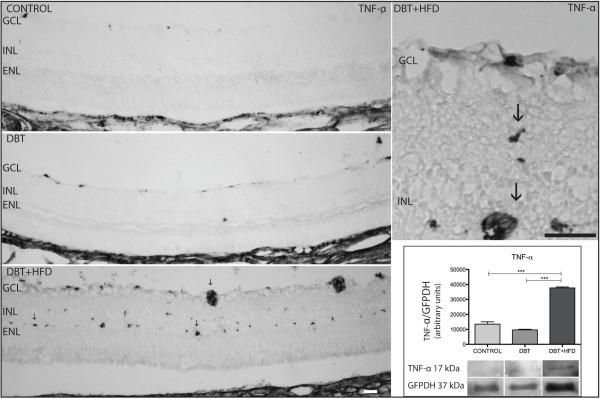
**TNF-α retinal expression.** The immunohistochemical expression of TNF-α was found to be upregulated in vessels of the inner retina of the diabetic group fed on a HFD (arrows) compared to diabetic group on a normal diet and controls. Barr 10 μm. Western blot confirms the higher expression in the diabetic group on a HFD. (***p < 0.0005).

Upregulation of TNF-α was observed in diabetic animals on a HFD. Immunoreactivity was found in the IPL and outer plexiform layer (OPL) (Figure 7). The WB revealed a higher expression of TNF-α in diabetic rats fed on a HFD than in diabetic rats fed on a normal diet and controls (Figure 7).

**Figure 8:**
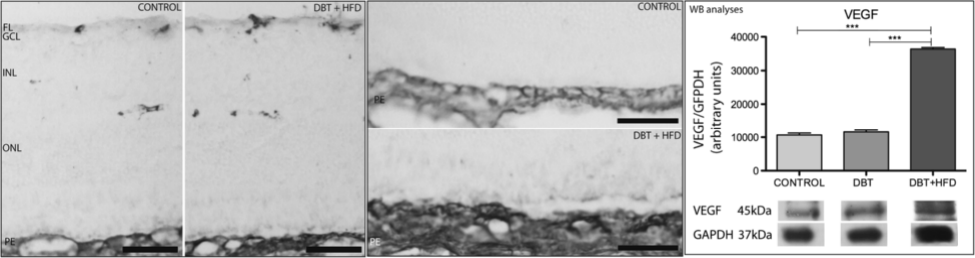
**Immunohistochemical analysis of VEGF expression in the different retinal layers.** A higher expression was found in diabetic rats on a HFD compared to controls. VEGF expression in the different layers: the fiber layer (FL), outer plexiform layer (OPL) and retinal pigment epithelium (RPE). Bar 50 μm. Western blot confirms the higher expression of VEGF in the HFD group. (***p < 0.0001).

VEGF was seen in the retina in the FL, OPL and RPE (Figure 8). WB analyses showed a higher expression of VEGF in rats fed on a HFD than in controls and diabetic rats on a normal diet (Figure 8).

**Figure 9:**
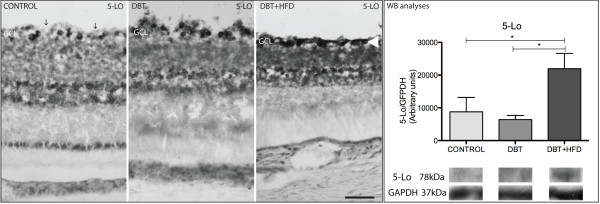
**Immunohistochemical analyses of 5-LO.** A similar immunohistochemical pattern was observed in the three groups of animals. The control group shows stained cells separated by free space (arrows) in the GCL while staining in the GCL of the HFD group is much more dense (arrow head). Bar 50 μm. The difference was confirmed by WB analyses. (*p < 0.05).

Immunoreactivity of 5-LO was observed in the GCL, in the INL and in the retinal pigment epithelium (RPE) of both diabetic rats and controls. Although there was a similar pattern among diabetic rats, staining extension was greater in diabetic animals on a HFD than in diabetic animals on a normal diet. In addition, a higher expression of 5-LO was observed on WB in animals fed on a HFD compared to controls and diabetic rats on a normal diet (Figure 9).

## Discussion

We have conducted an experimental study of diabetic retinopathy in diabetic rats fed on a HFD. In these animals inflammatory and proangiogenic molecules were found to be upregulated. To the best of our knowledge, this is the first study done in diabetic rats in which a HFD along with glycemia have a synergistic effect that leads to retinopathy development.

Most diabetic retinopathy studies carried out in rodents involve type 1 diabetes models. In this model the injury induced by the STZ injection causes complete destruction of pancreatic beta cells. This is different from what occurs in type 2 diabetes models in which beta cell loss takes place at a slower rate. However, the use of both a HFD and STZ in our diabetes model might confuse the reader. This model includes neonatal rats in which progenitor cells are still present in the pancreas after birth, inducing the regeneration of beta-cells and keeping up insulin secretion. Our model of diabetes was previously characterized by other investigators [12,16] and recommended for studies on diabetic complications associated with obesity [17].

Glycemia and triglyceridemia levels were found to be more elevated in diabetic animals than in controls. In our research we measured total cholesterol levels, but not low density and high-density lipoprotein levels. This might have yielded interesting data given the harmful effect of LDH on the cardiovascular system [18].

The fatty acid chemical compound diet in HFD animals shows a profile known to be risky for cardiovascular disease and favours cell damage [19-21]. Our results are consistent with this. In our group of animals on a HFD there was a significantly higher frequency of vessel abnormalities in the form of acellular capillaries and loss of pericytes as well as ganglion cells than in non-diabetic controls. Although several factors could have been involved in this unbalanced situation, we think that the decrease of docosahexaenoic acid-derived neuroprotectin D1 (NPD1) [22-24] was undoubtedly a major contributing factor. Arachidonic acid (AA) is catalyzed by the enzyme 5-LO, resulting in the production of platelet activating factor, prostaglandines, leukotrienes and thromboxanes, which are all metabolites involved in inflammatory diseases [25]. The biosynthesis of leukotrienes was found to be elevated in diabetic mice [26]. Leukotrienes increase vascular permeability and play a crucial role in activating the nuclear factor kappa B (NF-κB) proinflammatory pathway [27]. The 5-LO enzyme has been involved in diabetes and its complications [28]; for example, in cardiac ischemia reperfusion where the use of 5-LO RNAi showed protective effects [29]. In the retina, the 5-LO promotes the presence of lipid inflammatory mediators in ischemic disorders [30] and it has also caused retinal capillary degeneration in a diabetes mouse model [31]. In our study 5-LO immunoreactivity was identified in the RPE and GCL of diabetic animals. However, protein expression was found to be significantly higher in animals fed on a HFD. Interestingly, 5-LO has recently been discovered as a potential therapeutic target for ocular diseases associated with angiogenesis [32,33]. Moreover, zileuton, a specific 5-LOX inhibitor, may play a role in the reduction [34] of angiogenesis.

RAGE is known to be involved in diabetic retinopathy pathogenesis. In early diabetes advanced-glycation end products modulate retinal vasopermeability and alter interendothelial cell tight junction integrity, leading to inner blood retinal barrier dysfunction. This seems to result from the upregulation of VEGF [35]. RAGE ligation in endothelial cells activates the NF-κB. An increase of cells with activated NF-κB has been found in diabetic rats with RAGE-dependent regulation [36]. RAGE and its ligands are increased in the vitreous cavity of eyes with proliferative diabetic retinopathy, suggesting a role in the development of proliferative retinal diseases [28,37]. RAGE and VEGF may constitute a powerful association that hastens diabetic retinopathy.

Early in the course of diabetic retinopathy, Müller cells markedly upregulate the expression of GFAP [6], a nonspecific response to pathophysiological conditions. Müller cells produce factors capable of modulating blood flow, vascular permeability, and cell survival, and their processes surround all blood vessels in the retina. The role of these cells in the pathogenesis of retinal microangiopathy has therefore been established [38]. VEGF is known to be an essential agent in retinal inflammation, vascular leakage, hypoxia and angiogenesis. In our group of diabetic animals fed on HFD, VEGF immunoreactivity was observed in the fiber layer, outer plexiform layer and RPE layer. VEGF can be produced by vascular endothelial cells, glial and ganglion cells, pericytes and retinal pigment epithelium cells. Based on their morphology and retinal localization, VEGF immunoreactivity was identified in retinal pigment epithelial cells and probably also in endothelial and glial cells. In a research carried out in VEGF knockout mice investigators reported a significantly reduced expression of inflammatory biomarkers, a relatively low number of acellular capillaries, and less vascular leakage compared to a group of diabetic control mice [39]. A VEGF antagonist, the pigment-epithelium-derived-factor (PEDF), reduces the effect of VEGF [40]. In patients, bevacizumab, pegaptanib, and ranibizumab (antiVEGF agents) are intravitreously injected in diabetic retinopathy and exudative macular degeneration. Aflibercept (VEGF Trap Eye) will be soon incorporated into clinical use. Nevertheless, a longing acting drug with a less frequent need for injections has not been found yet. Anyway, it is of ophthalmic interest that susceptibility of tumor cells to antiVEGF agents is dissimilar [41], probably due to levels of VEGFR2. Resistant cases have been found in association with low levels of VEGFR2. To our knowledge, this has not been investigated in the eye. Ophthalmologists are aware of cases of diabetic macular edema and proliferative diabetic retinopathy that minimally respond to antiVEGF therapy. Drug delivery systems or nanoparticles functionalized with antiVEGF molecules could be used to work this out [42].

The role of the inflammatory cytokine TNF-α in the pathogenesis of diabetic retinopathy is well established. In recent investigations, retinal inflammation and apoptosis of microvascular cells and neurons were significantly reduced in the absence of TNF-α [43]. In rheumatoid arthritis and other systemic inflammatory diseases several TNF-α inhibitors have been used (adalimunab [humira], etanercept [enbrel], infliximab [remicade]) [44]. Cimzia is a new TNF-blocker, which shows less toxicity and a higher affinity to human TNF [45]. Actemra is a monoclonal antibody blocker of IL-6 that also affects TNF-α. The two TNF-α receptors, TNFR1 and TNFR2, have been found to be involved in ischemia reperfusion injury and would be interesting targets for pharmacological therapies [46]. Finally, a recent research study reported that TNF-α resistance promotes drug resistance in malignant tumors [47,48] posing an interesting hypothesis for analysis in eye research.

In a study done in humans on the profile of lipids and proteins with paracrine functions encountered in the vitreous, the progression of diabetic retinopathy correlated with increased levels of 5-LO metabolites and VEGF, which is to some extend similar to the findings of our study [49]. In our opinion several inflammatory and proangiogenic factors are involved in the development of diabetic retinopathy. However, most ocular treatments have included monotherapies. This approach contrasts with what is often implemented in oncology, that is to say, combined pharmacological therapies, targeting different sites to tackle several disease mechanisms [50]. We think that combined pharmacological therapies should be tested more frequently in experimental diabetic retinopathy studies.

## Conclusions

It is noteworthy that an injection of 45 mg/kg of STZ and a HFD resulted in much more retinal changes than a single STZ injection of 90 mg/kg in rats. These results support the hypothesis of a synergistic effect of lipotoxicity and glucotoxicity on the retina. The model of neonatal diabetic rats fed on a HFD seems to be useful to further evaluate mechanisms involved in diabetic retinopathy progression as well as to find new therapeutic targets.

## Abbreviations

HFD: High fat diet; GFAP: Glial fibrillary acidic protein; IRMA: Intra retinal microvascular alterations; STZ: Streptozotocin; VEGF: Vascular endothelial growth factor; RAGE: Receptor of advanced glycation end products; 5-Lo: 5 lipo-oxigenase; TNF-α: Tumor necrosis factor-alpha; GFPDH: Glyceraldehyde-3-phosphate dehydrogenase; GCL: Ganglion cell layer; IPL: Inner plexiform layer; INL: Inner nuclear layer; OPL: Outer plexiform layer; RPE: Retinal pigment epithelia; WB: Western blot; AA: Arachidonic acid.

## Competing interests

The authors declare that they have no competing interests.

## Authors’ contributions

JEM: AB, MT, ES, FG; GO: AB, MT; JC: MT, FG; JEG: MT, ES, FG. All authors read and approved the final manuscript.

## Pre-publication history

The pre-publication history for this paper can be accessed here:

http://www.biomedcentral.com/1471-2415/13/14/prepub

## References

[B1] Marx J Unraveling the causes of diabetes Science 2002 296 5568 686 689 10.1126/science.296.5568.686 11976439

[B2] Tataranni PA Bogardus C Changing habits to delay diabetes N Engl J Med 2001 344 18 1390 1392 11333998 10.1056/NEJM200105033441809

[B3] Sinha R Fisch G Teague B Tamborlane WV Banyas B Allen K Prevalence of impaired glucose tolerance among children and adolescents with marked obesity N Engl J Med 2002 346 11 802 810 10.1056/NEJMoa012578 11893791

[B4] Steinbrook R Facing the diabetes epidemic–mandatory reporting of glycosylated hemoglobin values in New York City N Engl J Med 2006 354 6 545 548 10.1056/NEJMp068008 16467539

[B5] Winer N Sowers JR Epidemiology of diabetes J Clin Pharmacol 2004 44 4 397 405 10.1177/0091270004263017 15051748

[B6] Li Q Zemel E Miller B Perlman I Early retinal damage in experimental diabetes: electroretinographical and morphological observations Exp Eye Res 2002 74 5 615 625 10.1006/exer.2002.1170 12076083

[B7] Chew EY A simplified diabetic retinopathy scale Ophthalmology 2003 110 9 1675 1676 10.1016/S0161-6420(03)00815-7 13129860

[B8] Yamada H Yamada E Higuchi A Matsumura M Retinal neovascularisation without ischaemia in the spontaneously diabetic Torii rat Diabetologia 2005 48 8 1663 1668 10.1007/s00125-005-1809-0 15977012

[B9] Bonner-Weir S Trent DF Honey RN Weir GC Responses of neonatal rat islets to streptozotocin: limited B-cell regeneration and hyperglycemia Diabetes 1981 30 1 64 69 10.2337/diabetes.30.1.64 6112177

[B10] Pascoe WS Storlien LH Inducement by fat feeding of basal hyperglycemia in rats with abnormal beta-cell function. Model for study of etiology and pathogenesis of NIDDM Diabetes 1990 39 2 226 233 10.2337/diabetes.39.2.226 2146180

[B11] Chen WJD Grant MB Esselman JE Busik JV Dyslipidemia-Induced Adhesion Molecule Expression in hRVE Cell Invest Ophthalmol Vis Sci 2003 44 11 5016 5022 10.1167/iovs.03-0418 14578429

[B12] Pascoe WS Jenkins AB Kusunoki M Storlien LH Insulin action and determinants of glycaemia in a rat model of type 2 (non-insulin-dependent) diabetes mellitus Diabetologia 1992 35 3 208 215 10.1007/BF00400919 1563580

[B13] Weir GC Clore ET Zmachinski CJ Bonner-Weir S Islet secretion in a new experimental model for non-insulin-dependent diabetes Diabetes 1981 30 7 590 595 10.2337/diabetes.30.7.590 6114005

[B14] Bradford MM A rapid and sensitive method for the quantitation of microgram quantities of protein utilizing the principle of protein-dye binding Anal Biochem 1976 72 248 254 10.1016/0003-2697(76)90527-3 942051

[B15] Kuwabara T Cogan DG Studies of retinal vascular patterns. I. Normal architecture. Arch Ophthalmol 1960 64 904 911 10.1001/archopht.1960.01840010906012 13755464

[B16] Fantus IG Chayoth R O’Dea L Marliss EB Yale JF Grose M Insulin binding and glucose transport in adipocytes in neonatal streptozocin-injected rat model of diabetes mellitus Diabetes 1987 36 5 654 660 10.2337/diabetes.36.5.654 3552798

[B17] Srinivasan K Viswanad B Asrat L Kaul CL Ramarao P Combination of high-fat diet-fed and low-dose streptozotocin-treated rat: a model for type 2 diabetes and pharmacological screening Pharmacol Res 2005 52 4 313 320 10.1016/j.phrs.2005.05.004 15979893

[B18] Gravina CF Bertolami M Rodrigues GH Dyslipidemia: evidence of efficacy of the pharmacological and non-pharmacological treatment in the elderly J Geriatr Cardiol 2012 9 2 83 90 10.3724/SP.J.1263.2011.12292 22916052 PMC3418895

[B19] De Lorgeril M Renaud S Mamelle N, al e. Mediterranean alpha-linoleic acid-rich diet in secondary prevention of coronary heart disease. Lancet 1994 343 1454 1459 10.1016/S0140-6736(94)92580-1 7911176

[B20] De Lorgeril M Salen P Martin JM Monjaud I Delaye J Mamelle N Mediterranean diet, traditional risk factors, and the rate of cardiovascular complications after myocardial infarction: final report of the Lion Diet Heart Study Circulation 1999 99 779 785 10.1161/01.CIR.99.6.779 9989963

[B21] Siscovick DS Raghunathan T King I Dietary intake of long-chain n-3 polynsaturated fatty acids and the of primary cardiac arrest Am J Clin Nutr 2000 71 208 212 10.1093/ajcn/71.1.208S 10617973

[B22] Bazan NG Survival signaling in retinal pigment epithelial cells in response to oxidative stress: significance in retinal degenerations Adv Exp Med Biol 2006 572 531 540 10.1007/0-387-32442-9_74 17249620

[B23] Bazan N Cell survival matters: docosahexaenoic acid signaling, neuroprotection and photoreceptors Trends Neurosci 2006 29 5 263 271 10.1016/j.tins.2006.03.005 16580739

[B24] Lukiw WJ Cui JG Marcheselli VL Bodker M Botkjaer A Gotlinger K A role for docosahexaenoic acid-derived neuroprotectin D1 in neural cell survival and Alzheimer disease J Clin Invest 2005 115 10 2774 2783 10.1172/JCI25420 16151530 PMC1199531

[B25] Samuelsson B Dahlen SE Lindgren JA Rouzer CA Serhan CN Leukotrienes and lipoxins: structures, biosynthesis, and biological effects Science 1987 237 4819 1171 1176 10.1126/science.2820055 2820055

[B26] Talahalli R Zarini S Sheibani N Murphy RC Gubitosi-Klug RA Increased synthesis of leukotrienes in the mouse model of diabetic retinopathy Invest Ophthalmol Vis Sci 2010 51 3 1699 1708 10.1167/iovs.09-3557 19834040 PMC2868429

[B27] Sanchez-Galan E Gomez-Hernandez A Vidal C Martin-Ventura JL Blanco-Colio LM Munoz-Garcia B Leukotriene B4 enhances the activity of nuclear factor-kappaB pathway through BLT1 and BLT2 receptors in atherosclerosis Cardiovasc Res 2009 81 1 216 225 10.1093/cvr/cvn277 18852255

[B28] Zong H Ward M Stitt AW AGEs, RAGE, and diabetic retinopathy Curr Diab Rep 2011 11 4 244 252 10.1007/s11892-011-0198-7 21590515

[B29] Lisovyy OO Dosenko VE Nagibin VS Tumanovska LV Korol MO Surova OV Cardioprotective effect of 5-lipoxygenase gene (ALOX5) silencing in ischemia-reperfusion Acta Biochim Pol 2009 56 4 687 694 20011686

[B30] Hardy P Beauchamp M Sennlaub F Gobeil F Jr Tremblay L Mwaikambo B New insights into the retinal circulation: inflammatory lipid mediators in ischemic retinopathy Prostaglandins Leukot Essent Fatty Acids 2005 72 5 301 325 10.1016/j.plefa.2005.02.004 15850712

[B31] Gubitosi-Klug RA Talahalli R Du Y Nadler JL Kern TS 5-Lipoxygenase, but not 12/15-lipoxygenase, contributes to degeneration of retinal capillaries in a mouse model of diabetic retinopathy Diabetes 2008 57 5 1387 1393 10.2337/db07-1217 18346986 PMC4444435

[B32] Stahl A Sapieha P Connor KM Sangiovanni JP Chen J Aderman CM Short communication: PPAR gamma mediates a direct antiangiogenic effect of omega 3-PUFAs in proliferative retinopathy Circ Res 2010 107 4 495 500 10.1161/CIRCRESAHA.110.221317 20634487 PMC2975617

[B33] Sapieha P Stahl A Chen J Seaward MR Willett KL Krah NM 5-Lipoxygenase metabolite 4-HDHA is a mediator of the antiangiogenic effect of omega-3 polyunsaturated fatty acids Sci Transl Med 2011 3 69 69ra12 10.1126/scitranslmed.3001571 21307302 PMC3711031

[B34] Rossi A Pergola C Koeberle A Hoffmann M Dehm F Bramanti P The 5-lipoxygenase inhibitor, zileuton, suppresses prostaglandin biosynthesis by inhibition of arachidonic acid release in macrophages Br J Pharmacol 2010 161 3 555 570 10.1111/j.1476-5381.2010.00930.x 20880396 PMC2990155

[B35] Kaji Y Usui T Ishida S Yamashiro K Moore TC Moore J Inhibition of diabetic leukostasis and blood-retinal barrier breakdown with a soluble form of a receptor for advanced glycation end products Invest Ophthalmol Vis Sci 2007 48 2 858 865 10.1167/iovs.06-0495 17251488

[B36] Shoji T Koyama H Morioka T Tanaka S Kizu A Motoyama K Receptor for advanced glycation end products is involved in impaired angiogenic response in diabetes Diabetes 2006 55 8 2245 2255 10.2337/db05-1375 16873687

[B37] Yamagishi S Nakamura K Matsui T Advanced glycation end products (AGEs) and their receptor (RAGE) system in diabetic retinopathy Curr Drug Discov Technol 2006 3 1 83 88 10.2174/157016306776637555 16712466

[B38] Mizutani M Gerhardinger C Lorenzi M Muller cell changes in human diabetic retinopathy Diabetes 1998 47 3 445 449 10.2337/diabetes.47.3.445 9519752

[B39] Wang J Xu X Elliott MH Zhu M Le YZ Muller cell-derived VEGF is essential for diabetes-induced retinal inflammation and vascular leakage Diabetes 2010 59 9 2297 2305 10.2337/db09-1420 20530741 PMC2927953

[B40] Zhang SX Wang JJ Gao G Shao C Mott R Ma JX Pigment epithelium-derived factor (PEDF) is an endogenous antiinflammatory factor FASEB J 2006 20 2 323 325 16368716 10.1096/fj.05-4313fje

[B41] Sitohy B Nagy JA Jaminet SC Dvorak HF Tumor-surrogate blood vessel subtypes exhibit differential susceptibility to anti-VEGF therapy Cancer Res 2011 71 22 7021 7028 10.1158/0008-5472.CAN-11-1693 21937680 PMC3217088

[B42] Stewart MW Rosenfeld PJ Penha FM Wang F Yehoshua Z Bueno-Lopez E Pharmacokinetic Rationale for Dosing Every 2 Weeks Versus 4 Weeks with Intravitreal Ranibizumab, Bevacizumab, and Aflibercept (Vascular Endothelial Growth Factor Trap-Eye) Retina 2012 32 3 434 457 22374154 10.1097/IAE.0B013E31822C290F

[B43] Huang H Gandhi JK Zhong X Wei Y Gong J Duh EJ TNFalpha is required for late BRB breakdown in diabetic retinopathy, and its inhibition prevents leukostasis and protects vessels and neurons from apoptosis Invest Ophthalmol Vis Sci 2011 52 3 1336 1344 10.1167/iovs.10-5768 21212173 PMC3101693

[B44] Kim IH West CE Kwatra SG Feldman SR O’Neill JL Comparative efficacy of biologics in psoriasis: a review Am J Clin Dermatol 2012 13 6 365 374 10.2165/11633110-000000000-00000 22967166

[B45] Weinblatt ME Fleischmann R Huizinga TW Emery P Pope J Massarotti EM Efficacy and safety of certolizumab pegol in a broad population of patients with active rheumatoid arthritis: results from the REALISTIC phase IIIb study Rheumatology (Oxford) 2012 51 12 2204 2214 10.1093/rheumatology/kes150 22923753

[B46] Gesslein B Hakansson G Gustafsson L Ekstrom P Malmsjo M Tumor necrosis factor and its receptors in the neuroretina and retinal vasculature after ischemia-reperfusion injury in the pig retina Mol Vis 2010 16 2317 2327 21152396 PMC2994763

[B47] Antoon JW White MD Burow ME Beckman BS Dual inhibition of sphingosine kinase isoforms ablates TNF-induced drug resistance Oncol Rep 2012 27 6 1779 1786 22469881 10.3892/or.2012.1743 PMC4028227

[B48] Seasholtz TM Chang J Smith J Walsh C Brown J Radeff-Huang j Tumor necrosis factor-alpha-stimulated cell proliferation is mediated through sphingosine kinase-dependent Akt activation and cyclin D expression J Biol Chem 2007 282 863 870 17114809 10.1074/jbc.M601698200

[B49] Schwartzman ML Iserovich P Gotlinger K Bellner L Dunn MW Sartore M Profile of lipid and protein autacoids in diabetic vitreous correlates with the progression of diabetic retinopathy Diabetes 2010 59 7 1780 1788 10.2337/db10-0110 20424229 PMC2889779

[B50] Xu T Chen J Lu Y Wolff JE Effects of bevacizumab plus irinotecan on response and survival in patients with recurrent malignant glioma: a systematic review and survival-gain analysis BMC Cancer 2010 10 252 10.1186/1471-2407-10-252 20525214 PMC2891637

